# Advances in cell therapy for solid tumours: European perspective and future directions

**DOI:** 10.1016/j.lanepe.2026.101590

**Published:** 2026-03-19

**Authors:** Victor Moreno, Fiona Thistlethwaite, Ramón Yarza, Willemijn S. Tak, Kok Haw Jonathan Lim, John B.A.G. Haanen

**Affiliations:** a START Madrid-FJD, Hospital Universitario Fundación Jimenez Diaz, 28040, Madrid, Spain; b Advanced Immunotherapy and Cell Therapy Team, Department of Medical Oncology, The Christie NHS Foundation Trust, Manchester, M20 4BX, UK; c Division of Cancer Sciences, Faculty of Biology, Medicine and Health, The University of Manchester, Manchester, M20 4GJ, UK; d START Madrid-CIOCC, Centro Integral Oncológico Clara Campal, 28050, Madrid, Spain; e Division of Medical Oncology, Netherlands Cancer Institute, Amsterdam, the Netherlands; f Department of Medical Oncology, Leiden University Medical Center, Leiden, the Netherlands; g Melanoma Clinic, Centre Hospitalier Universitaire Vaudois, Lausanne, Switzerland

**Keywords:** Cell therapy, Solid tumors, Tumor infiltrating lymphocytes (TILs), Chimeric antigen receptor T cells (CAR-T), T-cell receptor therapy (TCR-T), Advanced therapy medicinal products (ATMPs), Immunotherapy, European Medicines Agency (EMA), Gene

## Abstract

Cell therapy has revolutionised the landscape of cancer treatment, with therapies such as Chimeric Antigen Receptor T cells (CAR-T cells), showing remarkable efficacy in haematological malignancies, and approaches such as Tumour Infiltrating Lymphocytes (TILs) and T-cell receptor-engineered T cells (TCR-T) showing increasing promise in solid tumours. The recent US FDA approvals of lifileucel (a TIL therapy for advanced melanoma) and afamitresgene autoleucel (a TCR therapy targeting MAGE-A4 in synovial sarcoma) mark the first regulatory recognition of cell therapies for solid tumours and signal a new era for oncology. Europe has played a central role in these advances, leading pivotal phase 3 trials and pioneering hospital-exemption-based manufacturing programmes. However, the continent still faces major challenges, including fragmented regulatory frameworks, high manufacturing costs, and inequitable patient access across member states. Emerging innovations such as gene-edited, allogeneic, and iPSC-derived cell products promise to address current limitations by improving scalability, safety, and time-to-treatment. This Series paper examines the latest advancements in cell therapy, focussing on the European experience, while comparing global trends. We discuss challenges specific to Europe, such as regulatory frameworks, manufacturing scalability, and disparities in access. Emphasis is placed on emerging innovations like gene-edited and allogeneic therapies, as well as future directions for integrating cell therapies into mainstream oncology. We conclude with recommendations for overcoming barriers related to cost, toxicity management, and equitable access across Europe.

**Key messages**

• **What has changed?** The US Food and Drug Administration (FDA) has approved the first two cell therapies for solid tumours in 2024: lifileucel, a Tumour Infiltrating Lymphocyte (TIL) therapy for advanced melanoma; and afamitresgene autoleucel (afami-cel), a T-cell receptor (TCR) therapy targeting MAGE-A4, for metastatic synovial sarcoma. Afami-cel is currently under evaluation by the European Medicines Agency (EMA). In addition, there has been a positive European academia-led phase 3 trial with TIL therapy for advanced melanoma, as second-line treatment. Currently, only Denmark and the Netherlands provide access to TIL by institutional approval.
• **Key challenges.** Adoptive cell therapy faces challenges in manufacturing, scalability, and cost. Equitable access across Europe remains a pressing issue.
• **Most promising approaches.** Gene-edited allogeneic cell therapies represent an emerging approach with considerable potential in cancer treatment. Platforms such as iPSC-derived CAR-T/NK cells, CAR-NK, and mesenchymal stem cells offer scalable, off-the-shelf solutions. CRISPR/Cas9 and base editing enhance safety, persistence, and immune evasion, addressing key limitations of current autologous and allogeneic strategies.

**Search strategy and selection criteria**

A structured literature search was conducted following PRISMA principles using PubMed, EMBASE, and the Cochrane Library for English-language human studies published between January 2018 and July 2025. The search strategy involved a comprehensive review of databases such as PubMed, EMBASE, and Cochrane. Search terms included “CAR-T cells,” “TIL therapy,” “cell therapy in cancer,” “modified TCRs” and “adoptive cell therapy” with Boolean operators used to refine results. To ensure historical context, seminal pre-2018 studies (e.g., Rosenberg et al.) were added through citation tracking. The search was complemented by manual review of reference lists, conference abstracts (ESMO, ASCO, SITC), and grey literature, including EMA/CAT reports, FDA communications, European Commission and HTA documents, and EFPIA publications cited in this review. Studies were included if they reported clinical, regulatory, or policy data on adoptive cell therapy for solid tumours in a European context. Preclinical studies and opinion pieces without new data were excluded.

## Introduction

For over a decade, immunotherapy has revolutionised survival outcomes for patients with metastatic cancer and has been progressively adopted as standard of care for many subtypes of cancer. Specifically, immune checkpoint inhibitors (ICI), in particular the combination of anti-PD-1 with either anti-CTLA4 or anti-LAG3, have provided durable survival benefit for more than one in two patients with metastatic melanoma.^1^^,^^2^ However, there is significant heterogeneity in response between patients with similar diagnosis. High ICI efficacy rates, similar to those seen in melanoma, have not been observed in cancer subtypes such as sarcoma, pancreatic cancer, glioblastoma and others.

**Fig. 1:**
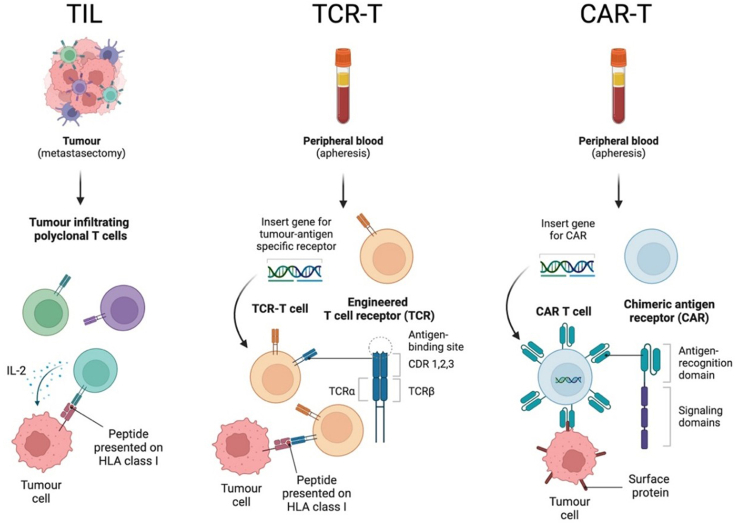
Generation and mechanism of current autologous T cell therapy agents, including tumour infiltrating lymphocytes (TILs), engineered T cell receptor therapy (TCR-T) and chimeric antigen receptor T cell therapy (CAR-T). Created with Biorender.com.

A potential limitation of ICI is the dependence on the intrinsic quantity and repertoire of, mostly exhausted, T cells *in vivo*. Cell-based therapies, whereby autologous immune cells are expanded at scale *ex vivo* prior to subsequent reconstitution in patients, such as tumour infiltrating lymphocytes (TILs) or T cell receptor engineered T cell (TCR-T) therapy (Fig. 1), have shown promising results in solid tumours including the potential for sustained long-term benefit. The feasibility of adoptive (living) cellular therapy was pioneered by Steven Rosenberg and colleagues at the US National Cancer Institute through their seminal work in the 1980's isolating native T cells from resected tumour tissue.^3^ In the first cohort of 20 patients with metastatic melanoma treated with adoptive transfer of TILs and high-dose interleukin-2 (IL-2), objective response was reported in 11 patients (55%), including regression in metastatic lesions in the lung, liver, bone and skin.^4^ This innovation laid the groundwork to subsequent clinical studies with TILs to treat common epithelial cancers, albeit the majority in the setting of small case series or early phase clinical trials, to varying levels of success.^5^^,^^6^

Subsequently, in the pursuit to improve precision of T cell therapy, Rosenberg's group introduced genetically-engineered T cells derived from peripheral blood in 2006, reporting the first safe use of TCR-T gene therapy targeting a melanoma-associated antigen MART-1.^7^ Here, individual patient's tumour-reactive T cells were generated by retroviral-transduction of CD8^+^ cytotoxic T cells to recognise HLA-A∗0201-bound MART-1 peptide, with objective response reported in n = 2/15 (13%) patients.^7^ Many other TCR-T are now in clinical development, ranging from those targeting tumour-associated tissue-differentiation antigens (e.g., MART-1, gp100, CEA) and cancer testis/germline antigens (e.g., NY-ESO-1, MAGE-A3, MAGE-A4, MAGE-A10, PRAME) to viral antigens and neoantigens.^8^

Concurrently, there has been the emergence of chimeric antigen receptor (CAR) T-cell therapy, spearheaded by successes in treating haematological malignancies. Similar to TCR-T, T cells are obtained by aphaeresis and are then genetically engineered—typically using viral vectors—to express CARs that target specific tumour-associated antigens (Fig. 1). Unlike TCRs, however, CARs recognise surface antigens independently of human leukocyte antigen (HLA), which allows for broader application and circumvents common immune evasion mechanisms, such as loss of antigen presentation machinery. Earlier generations of CAR-T faced issues with persistence and efficacy, and despite significant design improvements resulting in impressive outcomes in haematological malignancies, applying CAR-T to solid tumours has been much more challenging.^9^ This is in part due to significant tumour antigen heterogeneity, immunosuppressive tumour microenvironment, poor T cell trafficking and persistence, and risk of on-target/off-tumour toxicity.

In this series paper, we outline the current landscape of adoptive cell therapy (ACT) for solid tumours at the cutting edge of clinical application especially in the geography of Europe, and discuss opportunities, challenges and outlook of the roll out of this next-generation immunotherapy.

## Current landscape of cell therapy for solid tumours

In 2024, there was a breakthrough milestone with the approval of the first two cell therapy agents for solid tumours. Firstly, on the back of two parallel pivotal clinical trials of autologous unselected polyclonal TIL in previously pre-treated patients with advanced melanoma, lifileucel became the first cell-based advanced therapy medicinal product (ATMP) for solid tumours to be (accelerated) approved by the US Food and Drug Administration (FDA) in February 2024. In the global multicentre single-arm phase 2 C-144-01 registration trial (NCT02360579), lifileucel demonstrated an objective response rate (ORR) of 31%, with n = 8/153 (5%) patients achieving complete response (CR) (Table 1).^10^ Furthermore, lifileucel is currently undergoing evaluation in a pivotal phase 3 clinical trial as first line therapy in combination with pembrolizumab compared to pembrolizumab alone for patients with untreated, unresectable or metastatic melanoma in the first-line setting (NCT05727904).

**Table 1:** List of approved adoptive cell therapy for solid tumours.

Product	Type/Indication	Key results from registration trials	FDA status	EMA status
**Lifileucel**^10^ (AMTAGVI®), LN-144	TIL for advanced melanoma	• Phase 2 [NCT02360579], prospective, open-label, multicohort, multicentre—US, France, Germany, Hungary, Italy, Spain, Switzerland, UK. • Sample size: n = 153 • ORR 31% (rate of CR: 5%); mPFS 4.1 months; mOS 13.9 months; mDoR not reached.	Approved—February 2024	Application Withdrawn July 2025
**TM001**^11^ ∗*Special approval for the Netherlands and Denmark*	TIL for advanced melanoma	• Phase 3 [NCT02278887], prospective, open-label, randomised, two centres—Amsterdam, Copenhagen. • Comparator: Ipilimumab 3 mg/kg • Sample size: n = 168 (n = 84 TILs vs n = 84 Ipilimumab) • ITT: ORR 49% vs 21% (rate of CR: 20% vs 7%); mPFS 7.2 vs 3.1 months (HR 0.50, P < 0.001); mOS 25.8 vs 18.9 months.	n/a	Under review
**Afamitresgene autoleucel**^12^ (TECELRA®), ADP-A2M4	TCR-T (MAGE-A4) for unresectable/metastatic synovial sarcoma	• Phase 2 [NCT04044768], prospective, open-label, multicohort, multicentre—US, Canada, France, Spain, UK. • Sample size: n = 52 (n = 44 synovial sarcoma, n = 8 myxoid round cell liposarcoma. • Synovial sarcoma subgroup (n = 44): ORR 39% (all PR at best), mPFS 3.8 months; mDoR 11.6 months. Overall population mOS 15.4 months.	Approved—August 2024	Under review

*Abbreviations:* EMA, European Medicines Agency; CR, complete response; FDA, U.S. Food and Drug Administration; ITT, intention-to-treat; mDoR, median duration of response; mOS, median overall survival; mPFS, median progression-free survival; TCR-T, engineered T cell receptor therapy; PR, partial response; TILs, tumour infiltrating lymphocytes.

Meanwhile, in the landmark European study conducted at the Netherlands Cancer Institute (Amsterdam) and Herlev Hospital (Copenhagen), this first-of-its-kind phase 3 randomised clinical trial with TIL therapy (NCT02278887) showed a significantly improved ORR of 49% (CR in n = 17/84, 20%) compared to 21% with ipilimumab, in patients with advanced melanoma who had progressed on prior anti–PD-1 therapy.^11^ The median progression-free survival (PFS) was 7.2 months with TILs compared to 3.1 months with ipilimumab. Importantly, the safety profile for both studies were consistent with expectations, with most adverse events related to the lymphodepletion regimen and high-dose IL-2.

Simultaneously, the phase 2 SPEARHEAD-1 trial (NCT04044768) involving afamitresgene autoleucel (afami-cel), genetically-modified autologous T cells expressing the high-affinity T-cell receptors against the cancer/germline Melanoma-associated antigen A4 (MAGE-A4) loaded on HLA-A∗02, formed the basis for its FDA accelerated approval for the treatment of refractory unresectable/metastatic synovial sarcoma in August 2024. In this global multicentre single-arm registration trial, the ORR for the whole cohort was 37% (n = 17/44 synovial sarcoma, n = 2/8 myxoid round cell liposarcoma) (Table 1).^12^ Afami-cel demonstrated a manageable safety profile; the most common grade ≥3 treatment-related adverse events included cytopenias, infections, and cytokine release syndrome (CRS), which were largely reversible.

In addition, other early phase clinical trials are showing encouraging data of TCR-T cell therapy across common tumours. PRAME-directed IMA203 induced a 70% overall response rate (50% confirmed) in advanced melanoma at the recommended phase 2 dose, with responses lasting beyond a year and minimal high-grade toxicity, prompting a global phase 2/3 registrational trial (SUPRAME; NCT06743126). Also, CD8-enhanced MAGE-A4 T-cells (ADP-A2M4CD8) produced ∼40% responses in the SURPASS study (NCT04044859), including durable regressions in ovarian, head-and-neck, urothelial and gastro-oesophageal cancers, underscoring histology-agnostic potential.^13^^,^^14^

## Access to cell therapy in Europe

The Committee for Advanced Therapies (CAT) is the European Medicines Agency (EMA) committee responsible for the approval of ATMPs in the European Union (EU), including for cell and gene therapies. Overall, the rates of approval of ATMPs by the EMA are more conservative and lag in appraisal time frame, compared to the US FDA. For instance, as of early 2025, there have been a total of 20 ATMPs authorised in Europe compared to 44 in the US market.^15^^,^^16^ Although there is currently no cell therapy for a solid tumour approved by the EMA for standard of care use in Europe, TM001 and afami-cel are all undergoing active evaluation. EMA application for lifileucel was withdrawn in July 2025.

Meanwhile, patients with advanced cancer have been and will continue to have access to cell therapies via special local hospital or national approvals, or through participation in clinical trials (Table 1). In the context of standard of care settings, in the Netherlands, for example, patients with advanced melanoma have been able to access personalised TIL therapy with TM001 via a hospital exemption (HE) mechanism reimbursed by their basic health insurance coverage since January 2023, approved by the Dutch National Health Care Institute (Zorginstituut), as a continuum of the phase 3 TIL trial.^17^ This academia-coordinated point-of-care manufacture of TIL therapy has also enabled similar set up in Denmark, who also participated in the phase 3 TIL randomised controlled trial.

Individual countries in Europe have been focussing on national efforts to address regulatory complexities, enhance patient access, and maintain competitiveness in the global cell and gene therapy landscape. For instance, since 2018, the UK has formed a national Advanced Therapy Treatment Centre (ATTC) network to specifically address the unique and complex challenges of transitioning ATMPs from research settings to clinical applications and harmonise regulatory requirements for downstream implementation.^18^ More recently, in 2024, acknowledging significant breakthroughs and success in the field, Germany launched a National Strategy for Gene and Cell Therapies to streamline patient access to ATMPs and develop a strategic framework to establish interdisciplinary treatment centres.^19^

**Fig. 2:**
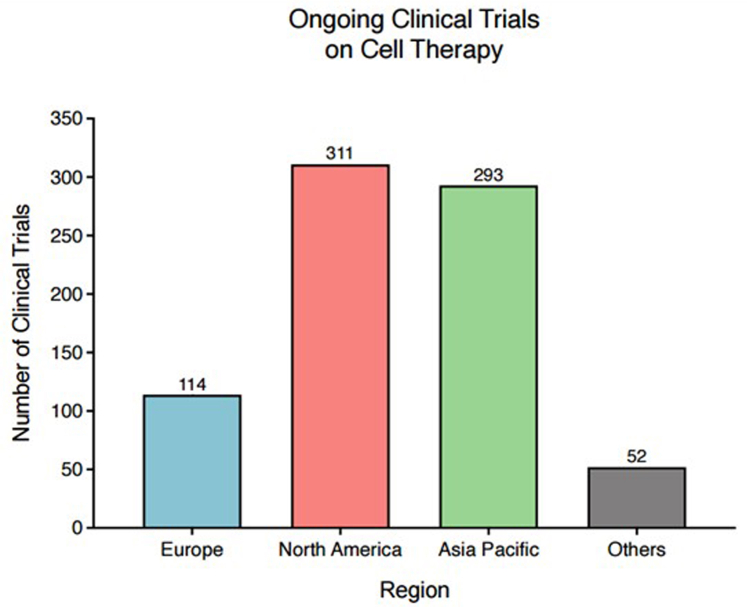
Ongoing clinical trials on cell therapy by region (July 2025). Ref (https://alliancerm.org/data/).

In summary, Europe remains a hub for clinical research in cell and gene therapies, with over 250 ongoing ATMP clinical trials as of early 2025.^20^ There are also emerging consortia-based efforts such as the multinational PragmaTIL project (12 partners, 6 countries), funded by the European Union (EU) Horizon Europe research and innovation programme, which aims to foster cross-institutional capability and capacity to accelerate the implementation of TIL therapy for patients.^21^ Nevertheless, it must be noted that there has been a steady decline in Europe's share of global cell and gene therapy trials since 2013 (from 22% to 12% in 2023),^22^ with major players like China showcasing exponential growth in this sector (Fig. 2).

## Challenges

Despite the promise of cell therapy for solid tumours, significant challenges remain which will impede its widespread adoption in Europe, including scientific and clinical hurdles, complex logistics, high costs, and fragmented regulatory and reimbursement systems.

### Biological barriers to successful cell therapy

In solid tumours, it is well-established that the tumour microenvironment is often immunosuppressive, characterised by dense stroma, undesirable metabolic conditions and hypoxia, and inhibitory cytokines that can impair T cell infiltration and function.^21^ Pervasive heterogeneity in (neo-)antigen expression further complicates effective therapeutic targeting, increasing the risk of tumour escape or disease recurrence. Therefore, it remains challenging to select the right epitopes/antigens to target, as many will be also shared with normal tissues, resulting in the significant risk of on-target, off-tumour toxicities.

## Concerns regarding serious toxicities and safety

Cell therapies, especially TCR-T and CAR-T, products, are associated with distinctive and sometimes severe toxicities. The most frequent are cytokine release syndrome (CRS) and immune effector cell–associated neurotoxicity syndrome (ICANS), which result from rapid immune activation and inflammatory cytokine release following TCR/CAR engagement and T cell activation. CRS typically presents within days following adoptive transfer with fever, hypotension, and hypoxia driven by IL-6, IFN-γ, and TNF-α release, whereas ICANS manifests as encephalopathy, seizures, or cerebral oedema and can occur independently of CRS.^23^ However, early data from lifileucel and afami-cel trials reported low rates of severe neurotoxicity, with most adverse events related instead to lymphodepletion or IL-2 administration.

In addition to immune-mediated toxicities, on-target/off-tumour effects remain a major safety concern in solid-tumour ACTs. Several early-phase trials encountered unexpected and sometimes fatal toxicities. Most notably, a fatality was reported in the 2010 trial of anti-HER2 CAR-T cells in a patient with metastatic colon cancer, who developed acute respiratory failure due to low-level HER2 expression in lung tissue, resulting in a cytokine storm.^24^ TCR-T therapies targeting MAGE-A3 have also led to severe off-target toxicities, including fatal neurological damage in two patients due to possible cross-reactivity with MAGE-A12 in the brain,^25^ and fatal cardiac toxicity in another trial due to T-cell recognition of the cardiac muscle protein Titin.^26^ Similarly, there have also been concerns raised in clinical trials targeting MART-1, with patients experiencing life-threatening complications,^27^ and in trials with TCR-T targeting CEA in colorectal cancer, resulting in severe inflammatory colitis due to antigen expression on healthy colonic epithelium.^28^

Emerging immune-related complications, notably immune effector cell–associated haemophagocytic syndrome (IEC-HS), have also been reported after CAR-T administration. Multidisciplinary management protocols incorporating early cytokine monitoring, IL-6 blockade, corticosteroids, IL-1R blockade, JAK1/2 inhibitors and supportive measures are being standardised to ensure rapid recognition and treatment.^29^ Collectively, these experiences underscore the need for robust safety oversight, rigorous pre-clinical antigen validation, and coordinated EU-wide pharmacovigilance to support the safe deployment of next-generation ACTs.

## Regulatory and policy landscape in Europe

**Fig. 3:**
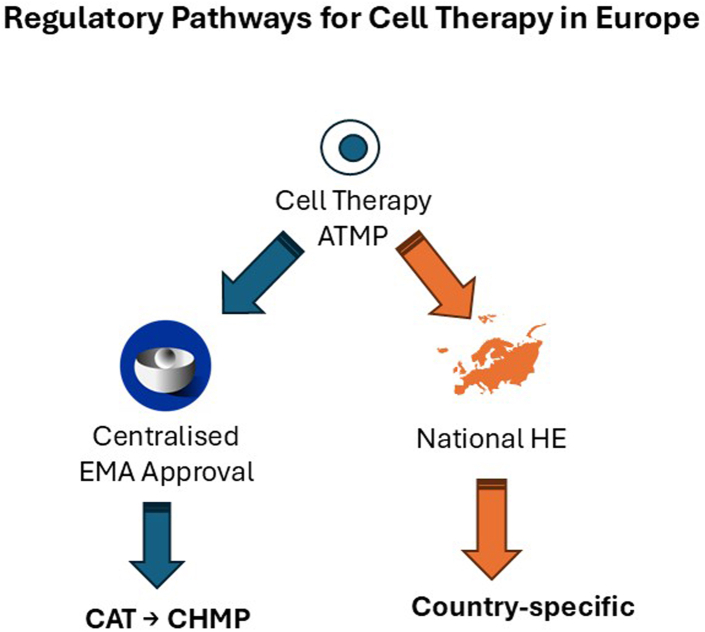
Schematic representation of different regulatory pathways in Europe. Advanced Therapy Medicinal Product (ATMP). Committee for Advanced Therapies (CAT). Committee for Human Medicinal Products (CHMP). Hospital Exemption (HE).

The European regulatory framework for ATMPs, including cell and gene therapies, is characterised by a complex interplay of national and European-level frameworks (Fig. 3), with the EMA, its CAT and Committee for Human Medicinal Products (CHMP) playing a pivotal role in the assessment, approval and facilitation of free movement of these products within the EU.^30^

Despite this centralised process, known challenges include regulatory differences across EU member states, varying HE and reimbursement practices, and complex logistics in manufacturing and delivering of cell therapies, which create inequity in access to cell therapies. EMA is responsible for the centralised marketing authorisation process of ATMPs through the Regulation (EC) No 1394/2007 legal framework. Although this regulation was designed to establish harmonisation of ATMP evaluation processes across EU member states, significant regulatory differences exist across European countries (Table 2).

**Table 2:** European regulatory heterogeneity for ATMPs.

Country/Region	Hospital Exemption (HE): scope & definition	Who authorises HE	Quantitative/“non-routine” limits	Clinical trial oversight (CTR)	Notable implementation notes	Refs
Netherlands	HE allowed; hospital-based manufacture for individual patients under physician responsibility	Dutch Health and Youth Inspectorate (IGJ)	Defined as non-routine; case-by-case volume controls	EMA CTR + national ethics; strong alignment with EMA/CAT	TIL therapy reimbursed since 2023; mature point-of-care pathways in NKI	https://english.cbg-meb.nl/
Germany	HE permitted with specific GMP and pharmacovigilance obligations	Paul-Ehrlich-Institut (PEI)	Explicit expectation of *small-scale/non-routine* output	EMA CTR + federal Länder ethics	National strategy (2024) to expand ATMP centres; robust GMP network	https://www.pei.de/EN/home/home-node.html
France	HE (ATU/early access replaced by new schemes) with strong central oversight	ANSM	Quantitative controls via protocol and site licencing	EMA CTR + CPP ethics	Early-access mechanisms dovetail with evidence development for ATMPs	https://ansm.sante.fr/
England (UK)	HE available; detailed MHRA/ATTC operational guidance	MHRA (operationally supported by ATTC network)	Practical limits through governance and monitoring	UK CTR equivalent; REC + MHRA	National ATTC network harmonises safety monitoring and data standards	https://www.gov.uk/government/organisations/medicines-and-healthcare-products-regulatory-agency
Spain	HE authorised at hospital level with AEMPS supervision	AEMPS + regional health authorities	No fixed numeric cap published; institution-specific limits	EMA CTR + CEIm ethics	Growing hospital-based programs; heterogeneous regional implementation	https://www.aemps.gob.es/
Italy	HE defined nationally with regional execution; hospital manufacture allowed	AIFA + regional authorities	Controlled via protocol and centre accreditation	EMA CTR + ethics	Strong experience with outcome-based agreements; active regional hubs	https://www.aifa.gov.it/en/home
Denmark	HE permitted; close collaboration with academic GMP facilities	Danish Medicines Agency (DKMA)	Non-routine expectation; small-scale batches	EMA CTR + ethics	Partner site for EU TIL pathways; efficient academic–clinical integration	https://laegemiddelstyrelsen.dk/en/
Sweden	HE possible; stringent GMP and reporting	Medical Products Agency (MPA)	Non-routine principle; site-level caps via licencing	EMA CTR + ethics	Centralised oversight; selective centre accreditation	https://www.lakemedelsverket.se/en
Poland (CEE exemplar)	HE framework less prescriptive; variable institutional experience	Office for Registration of Medicinal Products	Often undefined numeric limits; case-by-case	EMA CTR + ethics	Capacity and HTA timelines are primary barriers to rapid adoption	https://www.gov.pl/
EU	EMA/CAT does not grant HE; provides central scientific/marketing authorisation. HE is national.	–	“Non-routine” and patient-specific by design	EU CTR applies to interventional trials	Heterogeneity in HE definitions is a key barrier to harmonisation	https://www.ema.europa.eu/en/homepage

Abbreviations: AEMPS, Spanish Agency of Medicines; AIFA, Italian Medicines Agency; ANSM, French Medicines Agency; ATMP, advanced therapy medicinal product; ATTC, Advanced Therapy Treatment Centre; CAT, Committee for Advanced Therapies; CEIm, Spanish ethics committee; CBG/MEB, Medicines Evaluation Board (NL); CTR, Clinical Trials Regulation; DKMA, Danish Medicines Agency; GMP, good manufacturing practice; HE, Hospital Exemption; HTA, health technology assessment; IGJ, Dutch Health and Youth Care Inspectorate; MHRA, UK regulator; MPA, Swedish regulator; PEI, Paul-Ehrlich-Institut; REC, Research Ethics Committee; TILs, tumour infiltrating lymphocytes.

A major contributing factor to differences within Europe's regulatory landscape is the variation in HE programs. HE allows for ATMP production and application outside the centralised marketing authorisation pathway and is embedded in the EC No 1394/2007 legal framework. Under this provision, cell therapies can be produced on a non-routine basis at an individual patient level to provide therapy access to patients with severe, disabling or life-threatening diseases.^31^ National competent authorities (NCAs) in each member state are responsible for regulations considering ATMPs manufactured and administered under HE, leading to further regulatory discrepancies across Europe.^32^ Exacerbating this, ATMP-HE guidelines are interpreted differently by European countries due to a lack of clear definitions within these guidelines.^33^, ^34^, ^35^ For example, the “non-routine basis” that HE-ATMPs must be prepared on, including the maximum number of patients that can be treated and the quantity of ATMP products that may be produced under a single HE licence, has been shown to be interpreted differently across Europe.^36^ The MHRA in the UK and the Paul-Ehrlich-Institut in Germany set clear limits based on the scale and frequency of production, whereas countries such as Poland, Italy, and Spain provide no specific guidance. Only the Netherlands defines a maximum number of patients eligible under HE. This lack of common criteria contributes to significant differences in ATMP regulation and implementation across EU member states.

Differences in who may hold HE licences further add to regulatory variation across Europe. Most countries allow ATMP manufacturers to apply, whereas Spain restricts authorisation to hospitals, requiring each to file separate applications. This creates an institution-based rather than manufacturer-based system.^37^

Regulatory fragmentation across Europe largely stems from the decentralised evaluation of clinical trials. NCAs in each member state apply varying interpretations and timelines, resulting in inconsistencies in ATMP development and availability.^38^^,^^39^ To treamline harmonisation of clinical trials across the EU, the EMA introduced the Clinical Trials Regulation (CTR) in January 2022. Although national clinical trials will continue to be assessed by the member state in which the trial is conducted, multinational clinical trials now require collaboration between all member states involved.^40^ This fragmentation has fostered a landscape dominated by mid-sized, nationally based contract research organizations (CROs) whose primary strengths lie in regulatory expertise, linguistic fluency, and familiarity with local requirements. Furthermore, the EMA has recently issued a new guideline detailing the structure and data requirements for clinical trial applications involving investigational ATMPS, while also offering a perspective towards marketing authorisation approval.^41^ Together, these developments aim to enhance alignment in clinical trial regulations across the EU, reducing discrepancies between member states.

Therefore, although the current regulatory framework for ATMPs aims for harmonisation across Europe, it is heavily challenged by divergence caused by national-level regulations. Although the CTR represents a significant step towards regulatory convergence, it currently lacks the necessary details to fully achieve harmonisation. Nevertheless, several initiatives are underway to address these limitations. For instance, the CAT is creating an overview of national discrepancies and the European Commission has launched the Joint Action Increase NET programme to strengthen the regulatory capacity for NCAs through targeted training and knowledge exchange. Furthermore, platforms such as the EMA's Simultaneous National Scientific Advice facilitate early discussion between NCAs to ensure early aligntment of the opinions of different member states.^42^ Together, these efforts reflect a progressive shift towards a more coherent and integrated regulatory landscape, which is essential to reduce fragmentation across the EU.

### Economic challenges: reimbursement and manufacturing scale-up

Cell therapies are expensive to produce and administer, with list price for lifileucel (AMTAGVI®) marketed at US$563,000^43^ per patient and afami-cel (TECELRA®) at US$727,000^44^ per patient. Additional cost considerations for clinical visits, hospitalisation, lymphodepletion, and toxicity management will also need to be further considered. Academia-led point-of-care production at local institutes may drive the cost down to 15–20% of commercial products, for example for TIL therapy,^45^ but this will require significant upfront investment in infrastructure, governance and adequate skilled staff.

Costs of these therapies are covered differently, depending on whether they are produced commercially or non-commercially, such as those manufactured under HE. For commercial ATMPs, health technology assessment (HTA) bodies in each European member state independently decide on use, pricing and reimbursement of new health technologies including ATMPs following marketing authorisation by EMA.^46^ To account for the high prices of ATMPs, managed entry agreements (MEA), which are arrangements between manufacturers and payers that ensure access to reimbursement of health technologies, can be implemented. The two main models of MEAs are finance-based agreements and outcome based agreements.^47^ The former involves upfront payment, where the cost of therapy is covered either before or immediately after treatment, whereas with the latter, payment is contingent upon achieving predefined clinical outcomes, such as survival or complete response.^48^ Reimbursement models differ across Europe. Western countries, with larger healthcare budgets and established funding systems, can better absorb the costs of cell therapies (Supplementary Tables S1 and S2). In contrast, Eastern European nations often struggle to meet the high upfront expenses, despite potential long-term benefits such as fewer hospitalisations and longer survival. Although these differences are most evident between Western and Eastern, or high and low- and middle-income countries European countries, they also exist across more wealthy European countries. This can be illustrated by the reimbursement model applying to KYMRIAH® (tisagenlecleucel), a CAR-T cell therapy that was approved by the EMA to treat B-cell acute lymphoblastic leukaemia, diffuse large B-cell lymphoma and follicular lymphoma. In Germany, Italy and Spain, its reimbursement is managed through outcome based methods, where the manufacturer is required to provide a (partial) refund when agreed predefined outcomes are not met.^49^^,^^50^ To overcome challenges associated with ATMP reimbursement, there is a growing interest in developing non-commercial alternatives within academic and clinical institutions. Notable examples include ARI-0001 CAR-T cell therapy, developed by Hospital Clínic Barcelona for acute lymphoblastic leukaemia, and TIL therapy for advanced melanoma, developed by the Netherlands Cancer Institute, both of which have been approved under national HE programs. These institution-led initiatives aim to provide cost-effective alternatives to commercially available products by leveraging local expertise and resources, resulting in enhancement of accessibility and affordability for patients by reducing reliance on high-cost commercial therapies. However, non-commercial academic initiatives are sometimes viewed critically by pharmaceutical companies, due to concerns related to compliance with GMP standards, scalability of production and government support leading to potential unfair competition. Such concerns underscore the need for robust regulatory frameworks and transparent operational models to ensure product quality, ensure inclusive market conditions and promote long-term viability of academically developed ATMPs into healthcare systems.

In addition to reimbursement, manufacturing scale-up is another critical economic hurdle for cell therapies in Europe. Shifting from small-scale production in the context of clinical trials to commercial volumes produced in accordance with GMP has proven to be challenging and costly. ATMPs, are often developed in a patient-specific manner, contibuting to a complex, labour-intensive, manufacturing process often conducted in specialised facilities.^51^ Furthermore, the need for stringent quality controls, along with the logistical challenges of transporting patient-specific cells between different facilities, further drives up costs. The manufacturing of ATMPs can occur either centrally, with production taking place at a single location before global distribution, or locally, where the manufacturing process is conducted at hospitals or facilities within a specific region, each coming with organisational and operational challenges. While some Western European countries have invested in local manufacturing facilities to address aforementioned production challenges, others lack the infrastructure or expertise to do so, relying instead on centralised production hubs that may delay therapy delivery.^52^ Innovative solutions, such as allogeneic “off-the-shelf” cell therapies readily availble for patient treatment, have the potential to reduce costs, simplify manufacturing processes and increase access to cell therapies. Furthermore, standardising CAR-T cell production, for example by developing closed, automated systems, may facilitate manufacturing scale-up and further reduce costs.^53^

These solutions may facilitate point-of-care CAR-T cell therapy and ATMP production under HE, enabling hospitals to operate independently from the traditional pharmaceutical model. However, their widespread adoption will require significant investment in research, development, and infrastructure, as well as updated regulatory guidelines to accommodate novel production methods.

## Addressing disparities in access to ATMPs across Europe

Unequal access to cell therapies within Europe reflects broader regional inequities in cancer care, and with high prices of ATMPs and budget constraints, these are most evident between Western and Eastern European countries.^54^ In Eastern European countries lower healthcare spending, limited infrastructure and fewer clinical trials delay the adoption of cell therapies. Many countries lack accredited GMP-compliant manufacturing facilities that meet EMA standards, forcing reliance on external manufacturers and increasing both cost and logistical complexity.

In addition, variation in time-to-access across European countries further exacerbates these disparities. This aspect of access inequity is in part due to lengthy negotiations by HTAs on pricing and reimbursement following EMA marketing authorisation.^55^^,^^56^ This not only delays patient access, but may also have an impact on the commercial viability of ATMPs on certain markets, potentially discouraging manufacturers from persuing authorisation in less profitable regions. Although the EU transparency directive has set a deadline of 180 days for reimbursement decisions following marketing authorisation to facilitate timely access to ATMPs, it has been shown that time to access varies greatly.

**Fig. 4:**
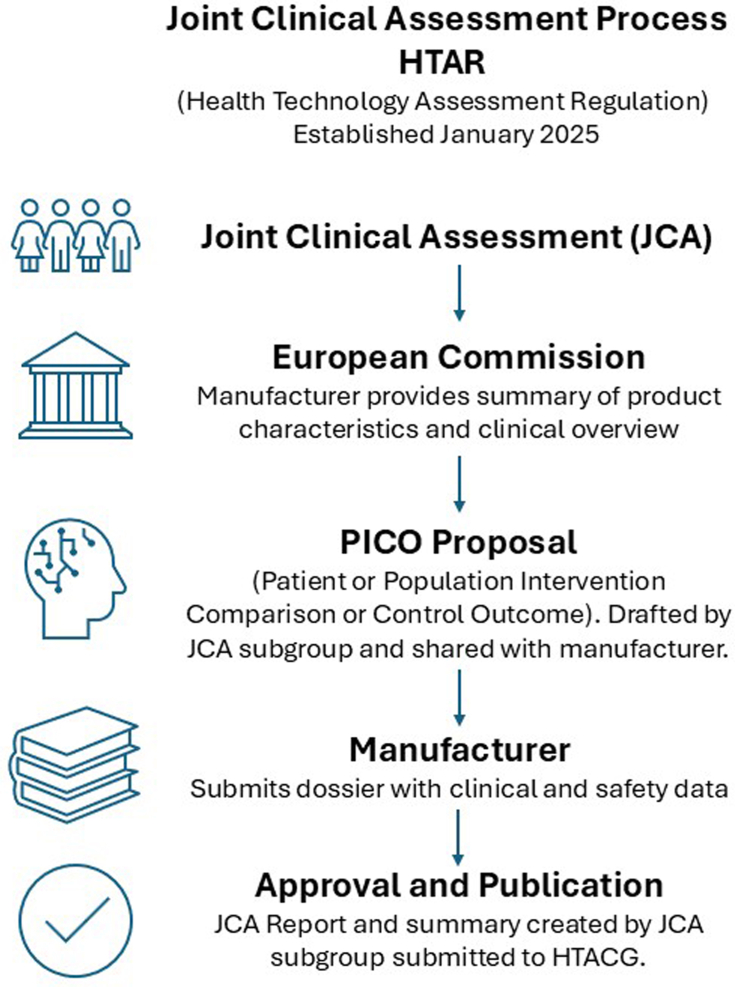
Joint clinical assessment process. “Under the new Health Technology Assessment Regulation (HTAR), Joint Clinical Assessments (JCAs) are coordinated by the JCA subgroup of the Member State Coordination Group on Health Technology Assessment (HTACG). ATMP manufacturers submit product summaries and clinical data alongside marketing authorisation applications.

In an effort to better streamline HTA across the EU to improve regulatory coordination and timely access, the Health Technology Assessment Regulation (HTAR), which establishes a framework for joint clinical assessments (JCA) of health technologies including ATMPs was brought into practice in January 2025 (Fig. 4).

National authorities are required to consider this JCA report in their own HTA processes and must inform the Health Technology Assessment Coordination Group (HTACG) of their final national assessments. Using this method, the EU aims to harmonise evaluations to ensure uniform decision-making across member states regarding the clinical effectiveness and safety of these technologies. Additionally, the HTAR accelerates patient access to innovative treatments, for example by requiring JCAs to be completed within 30 days of medicine's authorisation, reducing delays caused by varying HTA approaches across EU member states.

To address existing disparities in access to ATMPs, a multifaceted approach is required involving both policy-level reforms and collaborative initiatives. At the policy level, the EU could consider expanding programs like the Horizon Europe framework to provide funding and technical support for building cell therapy infrastructure in underserved regions. Collaborative initiatives, such as public-private partnerships and international consortia, could facilitate technology transfer, capacity building, and the establishment of regional manufacturing hubs.

## Academic vs industry-made products

Even when ACTs have transformed cancer treatment, significant differences exist between academic and industry-based approaches, influencing product characteristics, manufacturing, clinical applicability, and accessibility. For instance, industrial manufacturing involves large-scale, centralised production processes with stringent standardisation, quality control, and regulatory compliance. Products such as CD19-targeting tisagenlecleucel and axicabtagen ciloleucel represent examples of industry-standard CAR-T therapies with established manufacturing protocols, high costs, and long timelines due to complex logistics and regulatory demands.^57^ In contrast, academic institutions often use decentralised, point-of-care manufacturing characterised by higher flexibility, lower costs, and faster production cycles. However, academic manufacturing, despite its flexibility and innovation, has to comply with the same GMP requirements as larger pharmaceutical companies and is currently facing important barriers, including limited resources, regulatory limitations, and challenges achieving scalable production consistent with industrial standards.

To overcome such limitations, new strategies are ongoing to enhance the accessibility and efficacy of ACT based products. On the one hand, the development of automated, closed-system manufacturing platforms may reduce the complexity, time, and risk of contamination associated with manual production processes. Such an approach may enable efficient manufacturing at both centralised industrial and decentralised, point-of-care academic sites, significantly shortening times and lowering production costs. Concurrently, academic institutions are effectively adopting decentralised manufacturing practices within hospital units, producing freshly infused products.

Additionally, academia-industry partnerships are key to promoting further innovative approaches in the field. It is common that early-phase studies exploring novel constructs or targets may originate in academic settings and subsequently transition into industry-supported larger-scale production and commercialisation.^58^ In this sense, innovative engineering approaches must be pursued to further enhance therapeutic efficacy, safety, and applicability. To such approaches belong dual-targeting CAR constructs to mitigate tumour antigen escape, development of universal allogeneic CAR-T products to broaden patient eligibility, and innovative engineering strategies like CAR-NK cells and TCR constructs.

## Future directions

One of the primary advantages of cell therapy lies in its capacity to achieve durable responses in cancers that were once deemed refractory and even cure, as seen with CAR-T in haematological malignancies. However, the efficacy of these agents in the setting of solid tumours is tempered by biological barriers inherent to the tumour microenvironment and polyclonal nature of solid tumours.

Manufacturing remains a pivotal bottleneck in the widespread adoption of cell therapies. Today's therapies, primarily based on autologous models, rely on complex, individualised processes that require exceptional precision, rigorous quality control, and timely coordination for cell harvesting, genetic modification, and expansion. This personalised nature results in long production timelines and high costs. Decentralised, hospital-based manufacturing shows promise to alleviate some of these constraints by reducing costs through point-of-care production.^59^ However, significant investments in infrastructure, technological platforms (such as closed, automated bioreactor systems), and specialised training are required to achieve scalability while maintaining consistency across product batches. The emerging role of allogeneic “off-the-shelf” therapies, which could offer standardised, immediately available solutions, represents an exciting frontier that may eventually bypass some of the traditional obstacles of autologous manufacturing.^60^^,^^61^ Yet, these strategies are not without their own challenges, including potential immunological rejection and issues related to long-term cell persistence.

## Allogeneic cell therapies

Unlike autologous therapies, allogeneic therapies use universal donor cells, which enhances scalability, reduces costs, and enables faster availability. Induced pluripotent stem cells represent an example to this approach, offering a potentially unlimited source of standardised CAR-T, TCR-T and NK cells, which can be genetically tailored and manufactured efficiently in large-scale bioreactors.^62^

NK cells represent an important allogeneic therapeutic platform, demonstrating significant anticancer potential with important advantages such as absence of graft-vs-host disease (GvHD), rapid production, and broad applicability against both haematologic and solid tumours.^63^ Moreover, such models, including CAR-NK constructs, have shown promising early-phase clinical outcomes.^64^ On the other hand, mesenchymal stem cells, derived from umbilical cord sources, have shown to present immunomodulatory properties and therapeutic versatility, including tissue regeneration and anti-inflammatory effects. Such models are capable of evading immune detection through different mechanisms, making them attractive candidates for a wide range of clinical indications.

In addition to NK-based therapies, other innate immune cell platforms such as monocytes and macrophages are being engineered with chimeric antigen receptors (CAR-M) to enhance tumour phagocytosis and antigen presentation. CAR-M therapies have shown preclinical efficacy in reshaping the immunosuppressive tumour microenvironment and promoting adaptive immune activation. Early-phase clinical trial using first-in-class autologous CAR-M targeting HER2 (CT-0508) has demonstrated acceptable safety in patients with advanced solid tumours, although no partial responses were seen.^65^ Beyond HER2, early signals with mesothelin-targeted CAR-M in ovarian cancer support the platform's portability to additional antigens, although current information is limited to case reports.^66^ Ongoing optimisation includes next-generation CAR-M designs (e.g., poly-costimulatory domains, cytokine-secretion modules) intended to sustain *in vivo* activity and synergise with checkpoint blockade, with a CT-0508 + pembrolizumab cohort now underway.^67^ Other innovative cell types, including γδ T cells, invariant natural killer T (iNKT) cells, and macrophage-derived extracellular vesicles, are also under active exploration as alternative or complementary adoptive platforms to broaden the clinical scope of cell therapy in solid tumours.

Additionally, novel platforms such as invariant natural killer T cells and platelet-derived extracellular vesicles (EVs) can also enrich the therapeutic potential of allogeneic cell therapies. To this matter, also NKT cells, which lack alloreactivity, can be engineered with CAR constructs, providing enhanced tumour targeting and immune modulation.^68^

Nevertheless, critical challenges remain unsolved, including immune rejection, limited *in vivo* persistence, and manufacturing scalability. To overcome these, advanced gene-editing technologies, particularly CRISPR-based methods, are employed to eliminate immunogenic elements such as TCRs and HLA molecules, to mitigate rejection risks and enhance therapeutic longevity.

## Gene-edited cell therapies

Gene editing is becoming increasingly important in advancing ACTs, helping to overcome classic limitations of autologous approaches while improving the safety and versatility of engineered cell therapies. Among such technologies, CRISPR/Cas9 (*Clustered Regularly Interspaced Short Palindromic Repeats/CRISPR-associated protein 9*), TALENs (*Transcription Activator-Like Effector Nucleases*), and ZFNs (*Zinc Finger Nucleases*) have been successfully tested, allowing to delete, insert, or replace genetic material to improve immune cell performance in cancer therapy.^69^

CRISPR/Cas9 allows precise, multiplex gene knockouts to address key challenges in ACT, such as immune checkpoint resistance, GvHD, and host-vs-graft (HvG) rejection. Editing of genes like PD-1, CTLA-4, CD52, and TRAC has shown to preliminarily enhance T cell persistence and to reduce exhaustion.^70^ As an example, T cells edited to knock out SOCS1 or PD-1 have demonstrated improved antitumour activity in preclinical and early clinical trials.^71^

GvHD represents an important limitation in several cell therapy platforms. To reduce this risk, targeted deletion of the TRAC gene (TCRα constant region) has become a widely adopted strategy across multiple clinical platforms to remove endogenous TCR expression and prevent GvHD. For HvG evasion, strategies such as B2M knockout to eliminate HLA-I expression, combined with overexpression of HLA-E or CD47, have demonstrated promising *in vivo* persistence and reduced risk of rejection or NK-mediated lysis.

On the other hand, base-pair editing offers a high-precision alternative to traditional CRISPR, which enables gene disruption without inducing double-stranded breaks (DSBs). This approach has shown reduced genotoxicity with lower off-target risk, and successful multiplexing, as demonstrated in early-phase trials using CD7-targeted CAR-Ts.^72^ Meanwhile, alternative editing systems like Cas12a and Cas-Clover further increase the options for safe and effective multiplex genome modification. Looking forward, the field is moving toward finely edited, multi-layered gene control systems. Innovations such as miRNA-based regulation, synthetic switches, and modular shRNA platforms enable adjustable expression rather than full gene knockout, providing safer and more flexible control of engineered cell products.

However, despite remarkable progress, gene-edited cell therapies continue to raise safety concerns. One of the main issues is the risk of unintended genomic alterations, including chromosomal rearrangements, large deletions, and translocations particularly observed with nucleases that induce DSBs. Given that these genotoxic events may compromise cell function or introduce oncogenic risk, recent FDA advisories have pointed out these concerns, emphasising the need for enhanced genotoxicity screening and long-term follow-up in clinical trials using genome-edited products. Moreover, the regulatory landscape is evolving to incorporate specific guidance on gene editing, specifically regarding allogeneic and multiplex-edited products. As a result, developers are increasingly exploring safer alternatives such as base-pair editing or prime editing, which aim to minimise DSB-related toxicity while maintaining editing precision. Early clinical studies using these newer editors are ongoing and may help address some of the current safety limitations in gene-edited ACTs.

## Future regulatory and economic aspects

Regulatory frameworks and reimbursement models across Europe further complicate the current landscape. Despite the centralised role of the EMA in evaluating ATMPs, significant heterogeneity persists across member states. Variations in national interpretations of HE rules, clinical trial approvals, and manufacturing guidelines result in uneven access and prolonged timelines for patient access in many regions.^73^ While Western European countries have gradually streamlined processes and even piloted innovative reimbursement models such as pay-for-performance agreements, Eastern European nations and low- and middle-income countries within the continent still face significant obstacles.^74^

In addition to the regulatory and manufacturing challenges, the economic dimension of cell therapies must be addressed to guarantee their sustainability as a treatment modality. High production costs, compounded by additional expenses such as hospitalisation, supportive care, and toxicity management, place immense strain on national healthcare systems. Innovative reimbursement models that account for long-term benefits—such as reduced hospitalisation rates, improved quality of life, and increased productivity—are essential to justify the upfront investments. The development of MEAs and outcome-based reimbursement strategies can help bridge the gap between innovation and affordability, ensuring that the transformative potential of these therapies does not remain restricted to high-income settings but reaches all patients in need.^75^

Looking ahead, the future of cell therapy for solid tumours is both promising and complex. Ongoing advances in gene-editing technologies, including CRISPR/Cas9, TALENs, and emerging base-editing approaches, are set to enhance the safety and efficacy of engineered cell products. These techniques provide the precision needed to target tumour cells more effectively while minimising off-target effects and reducing toxicities. Moreover, the convergence of academic ingenuity with industry-scale manufacturing holds substantial promise for rapid innovation. Academia-industry partnerships can accelerate the translation of novel constructs from early-phase studies into commercial-grade products that meet stringent regulatory standards while also being economically viable.

## Conclusion

In conclusion, cell therapy for solid tumours has reached a pivotal moment, with TIL and TCR-based products demonstrating efficacy and gaining regulatory traction. Yet, the path to widespread clinical adoption in Europe remains constrained by complex biological, manufacturing, regulatory, and economic challenges. To create a more equitable and sustainable landscape, several strategic priorities must be pursued. Harmonising regulatory practices across EU member states—particularly for clinical trial approvals, HE, and GMP compliance—is critical to streamline access and reduce disparities. Innovative reimbursement models, such as outcome-based or pay-for-performance agreements, can align costs with clinical value and support broader adoption. Investment in decentralised GMP-compliant manufacturing, especially in underserved regions, will reduce costs and logistical barriers; closed, automated systems and off-the-shelf products can further accelerate scalability. Increased research funding, particularly through EU-level grants such as Horizon Europe, should support the development of scalable, off-the-shelf allogeneic therapies that can overcome autologous production bottlenecks. Capacity building and workforce training, through collaborative programs like Joint Action Increase NET, are essential to ensure pan-European expertise. Finally, global collaboration should be expanded to support technology transfer, regulatory convergence, and public–private partnerships—empowering all EU nations to adopt advanced therapies effectively. If addressed collectively, these actions will enable Europe to lead in delivering cell therapies that are not only innovative but also accessible, affordable, and equitable for patients with solid tumours.

**Recommendations**

To create a more equitable and sustainable landscape for cell therapies in Europe, several key strategies should be prioritised:

• **Harmonising regulatory practices**: Greater alignment of regulatory frameworks across EU member states is necessary to better streamline approval processes. Establishing centralised frameworks by the European Committee for clinical trial assessment and approval, HE guidelines and GMP compliance that apply across all European nations can enhance efficiency and ensure consistent quality standards, thereby reducing discrepancies.
• **Innovative reimbursement models:** Adopting outcome-based reimbursement models, such as pay-for-performance agreements, could help mitigate the financial burden of cell therapies. These models would tie payments to demonstrated clinical benefits, encouraging broader adoption while ensuring value for healthcare systems.
• **Investment in manufacturing infrastructure**: Expanding local GMP-compliant manufacturing facilities, particularly in countries with limited access, would reduce reliance on external production centres, lower costs and address logistical challenges. Development of closed, automated systems or off-the-shelft products may contribute to the decrease in preparation time and lead to further cost reductions, thereby facilitating ATMP manufacturing and adoption in less affluent countries. In addition, (international) public-private partnerships could play a pivotal role in funding these initiatives and operationalising regional hubs for these facilities.
• **Capacity building and training**: Developing a skilled workforce to support cell therapy research, manufacturing, and clinical implementation is critical. Collaborative training programs and knowledge-sharing initiatives, such as the Joint Action increaseNET, can help bridge the gap in expertise between Western and Eastern Europe.
• **Global collaboration**: Expanding European initiatives to extend the benefits of cell therapies across European countries and enable less affluent countries to adopt ATMPs more efficiently. These initiatives should focus on technology transfer, regulatory harmonisation, innovative reimbursement strategies, infrastructure investment and capacity building to ensure that the transformative potential of cell therapies is realised equitably and sustainably across Europe.

## Contributors

Concept and design: VM. Interpretation of the Data: VM, FT, RY, WT, KHJL, JBH. Writing of the Draft: VM, FT, RY, WT, KHJL, JBH. Approval of the final version: VM, FT, RY, WT, KHJL, JBH.

## Declaration of generative AI and AI-assisted technologies in the writing process

During the preparation of this work the authors used **ChatGPT (OpenAI, San Francisco, CA)** for language refinement of the manuscript. After using this tool, the authors reviewed and edited the content as needed and take full responsibility for the content of the publication.

## Declaration of interests

VM: Consulting fees from: Abbvie, Roche, Bayer, BMS, Janssen, Syneos, Affimed, Astra Zeneca, Merck, Ellipses Pharma. Principal Investigator—Institutional Funding: Abbvie, Achilles, Adaptimmune, Adc Therapeutics, Ascendis Pharma, Astrazeneca, Bayer, Beigene, Bicycle Tx, Bioinvent, Biomea Fusion, Biontech, Bms, Boehringer, C4 Therapeutics, Calico Life Sciences Llc, Celgene, Constellation, Crescendo Biologics, Cullinan, Daiichi Sankyo, Debiopharm, Dragonfly, Enliven Therapeutics, Epizyme, Exelixis, Famewave, F-Star Beta Limited, Genentech, Genmab, Gilead, Grey Wolf Therapeutics, Gsk, Hexal Ag & Sandoz, Hifibio, Hookipa Biotech, Hutchmed, Igm Biosciences, Imcheck Therapeutics, Immunocore, Immutep, Incyte Iomx Therapeutics, Iovance, Italfarmaco, Iteos, Janssen, Light Chain Bioscience, Lilly, Loxo Oncology, Merck, Merus, Miltenyi Biomedicine, Monta Biosciences, Msd, Mythic Therapeutics, Ningbo Newbay, Novartis, Oxford Biotherapeutics, Pfizer, Pharmamar, Pmv Pharma, Prelude Therapeutics Inc, Pyxis Oncology, Regeneron, Relay Terapeutics, Repare Therapeutics, Revolution, Roche, Schrödinger, Scorpion Therapeutics, Seagen, Shattucks, Synthorx, Takeda, Tango Therapeutics, Tesaro, Totus Medicines, Turning Point Therapeutics, Vividion Therapeutics.

FT: Consulting Fees from: AstraZeneca, Grey Wolf Therapeutics, Guidepoint, Immatics, OncoBayes, T-Knife Therapeutics, Waypoint. Principal Investigator—Institutional Funding: Achilles Ltd, Adaptimmune, Agalimmune limited, Amgen, Biontech, BMS, Chugai, Crescendo Biologics, GenMab, Grey Wolf Therapeutics, GSK, Immunocore, Incyte, Iovance, Janssen, Kymab Ltd/Sanofi, Leucid, Moderna, Novalgen, Nucana, Oxford Vacmedix, Roche, RS Oncology LLC, Seagen, Takeda, T-Knife, UCB, Zymeworks Other: Steering committee member for ATTC (Advanced Therapy Treatment Centre) Network.

RY: Principal Investigator—Institutional Funding: Abbvie, Ascendis Pharma, Bayer, Biontech, Boehringer, BMS, Daiichi Sankyo, Debiopharm, Eikon Therapeutics, GSK, Kura Oncology, Kumquat, Merck, Novartis, Nuvalent, OnKure, Pfizer, Pierre Fabre, Pyxis Oncology, Seagen.

WT: no competing interest to declare.

KHJL: Deputy lead of the MANIFEST consortium, which is core funded by the UK Government's Office for Life Sciences and the Medical Research Council (Grant Ref: MR/Z505158/1), with additional in-kind and/or financial contribution from IMU Biosciences, Guardant Health, Natera Inc., invoX Pharma Ltd., Genomics England, Northern Health Science Alliance (NHSA), M:M Bio, 10× Genomics, Roche, and Oxford Nanopore Technologies.

JBH: has participated in advisory board meetings with Agenus, Achilles Tx, AZ, BMS, BioNTech, CureVac, Eisai, EverImmune, GSK, Imcyse, Immunocore, Instil Bio, Intellia, Iovance Bio, Ipsen, Medigene, Merck Serono, Medigene, Molecular Partners, MSD, Neogene Therapeutics, Novartis, Orgenesis, Pfizer, Roche/Genentech, Sanofi, Sāstra, Third Rock Ventures, T-Knife and Tzu, received research grants from Amgen, Asher Bio, BioNTech US, BMS, MSD, Novartis and Sāstra, and has ownership interest in Neogene and Sāstra.
