# Supplementary material for: Advances in cell therapy for solid tumours: European perspective and future directions
Source: Lancet Reg Health Eur. 2026 Mar 19;64:101590. doi: 10.1016/j.lanepe.2026.101590 (PMC13147851; doi:10.1016/j.lanepe.2026.101590)
Supplement: Supplementary Table S1 [file mmc1.docx]

**Supplementary Table 1**. Reimbursement and access models for ATMPs in Europe (illustrative contrasts)

| **Country/Region** | **Primary HTA / pricing bodies** | **Dominant reimbursement model for ATMPs** | **Managed-access tools in use** | **Typical data expectations (post-launch)** | **Notes** |
| --- | --- | --- | --- | --- | --- |
| Netherlands | Zorginstituut (ZIN) + Ministry of Health | Outcome-linked coverage; selective central funding | CED | RWD registries; survival/response/HRQoL | TIL covered via basic insurance since 2023 |
| Germany | G-BA + IQWiG; PEI informs science | Statutory reimbursement; outcome-based elements via contracts | AMNOG framework; performance-linked rebates | RWD + registries; hospital add-ons | Early hospital funding mechanisms help ATMP uptake |
| France | HAS (CT/CEESP) + CEPS | CED with price–volume adjustments | Early access schemes; conditional funding | RWD via national platforms; periodic reassessment | Strong linkage of price to added clinical value |
| England (UK) | NICE + NHS England | Managed Access Agreements; outcomes-based | Cancer Drugs Fund / Innovative Medicines Fund | RWD through Blueteq/registries; survival endpoints | ATMPs often routed via specialised centres |
| Spain | Inter-territorial Council; CIPM pricing; regional HTA (RedETS) | Pilots for outcomes-based agreements; regional variability | Risk-sharing contracts in select regions | RWD obligations vary by region | Regional heterogeneity influences speed of access |
| Italy | AIFA + regional HTA | Broad use of outcomes-based (pay-for-performance, payment-by-results) | Robust national registries | Mandatory RWD capture; milestone-based payments | Longstanding experience with outcomes-based contracts |
| Denmark | Amgros + Medicinrådet | Central negotiation; selective outcomes-linked use | Time-limited conditional access | RWD in national health registries | High-quality registry infrastructure |
| Sweden | TLV + regional councils | Value-based; regional co-funding | National agreements + registry follow-up | Strong RWD via quality registers | Decentralised but data-rich |
| Poland | AOTMiT + MoH | Predominantly budget-impact driven; limited outcomes-based | Pilot risk-sharing agreements | Focused RWD where feasible | Budget impact and infrastructure are main constraints |

*Indicative timing reflects post-authorisation negotiation to first funded use in specialised centres and will vary by product, indication, and evidence maturity.

CED: Coverage with Evidence Development – Reimbursement model granting conditional coverage while additional real-world data are collected.

RWD: Real-World Data – Observational data collected from routine clinical practice to supplement trial evidence.

HRQoL: Health-Related Quality of Life – Patient-reported outcome metric used in HTA and cost-effectiveness evaluations.
