# Supplementary material for: Advances in cell therapy for solid tumours: European perspective and future directions
Source: Lancet Reg Health Eur. 2026 Mar 19;64:101590. doi: 10.1016/j.lanepe.2026.101590 (PMC13147851; doi:10.1016/j.lanepe.2026.101590)
Supplement: Supplementary Table S2 [file mmc2.docx]

**Supplementary Table 2.** European Regulatory Agencies involved in Reimbursement process.

| Abbreviation | Full Term | Explanation / Role |
| --- | --- | --- |
| AIFA | Agenzia Italiana del Farmaco (Italian Medicines Agency) | National authority responsible for the regulation, pricing, and reimbursement of medicines in Italy. |
| AOTMiT | Agencja Oceny Technologii Medycznych i Taryfikacji (Agency for Health Technology Assessment and Tariff System, Poland) | Poland’s national HTA and pricing authority responsible for evaluating medical technologies and setting reimbursement tariffs. |
| Amgros | Amgros I/S | Denmark’s national procurement organisation that negotiates drug prices for the Danish regions and hospitals. |
| CEESP | Commission d’Évaluation Économique et de Santé Publique | French Economic and Public Health Evaluation Committee under HAS; conducts economic evaluations of health technologies. |
| CEPS | Comité Économique des Produits de Santé | French Economic Committee for Health Products; sets drug prices after HAS evaluation. |
| CIPM | Comisión Interministerial de Precios de los Medicamentos | Spain’s inter-ministerial pricing commission that determines national drug prices following HTA input. |
| CT | Commission de la Transparence | French Transparency Commission (part of HAS) that assesses clinical benefit and improvement over existing therapies. |
| G-BA | Gemeinsamer Bundesausschuss (Federal Joint Committee, Germany) | Germany’s highest decision-making body for statutory health insurance; decides on reimbursement of medicines and medical procedures. |
| HAS | Haute Autorité de Santé (French National Authority for Health) | Independent public body that performs clinical and economic evaluations of health technologies in France. |
| HTA | Health Technology Assessment | Evidence-based evaluation process used by national bodies to determine clinical and economic value of health technologies. |
| IQWiG | Institut für Qualität und Wirtschaftlichkeit im Gesundheitswesen (Institute for Quality and Efficiency in Health Care, Germany) | German institute that provides scientific assessments of medical treatments and technologies to support G-BA decisions. |
| NICE | National Institute for Health and Care Excellence (United Kingdom) | Public body providing national guidance and recommendations on clinical effectiveness and cost-effectiveness of therapies. |
| PEI | Paul-Ehrlich-Institut (Federal Institute for Vaccines and Biomedicines, Germany) | German federal agency responsible for the evaluation and approval of biologics and ATMPs. |
| TLV | Tandvårds- och läkemedelsförmånsverket (Dental and Pharmaceutical Benefits Agency, Sweden) | Swedish government agency assessing cost-effectiveness and determining reimbursement for medicines. |
| ZIN | Zorginstituut Nederland (Dutch National Health Care Institute) | National body that advises on the inclusion of medicines in the basic health insurance package in the Netherlands. |
